# Genomic and Pathogenic Characterization of a Novel Capsule-Deficient Neonatal Meningitis-Associated *Escherichia coli* from Calves

**DOI:** 10.3390/vetsci13040401

**Published:** 2026-04-19

**Authors:** Jinchun Cai, Borui Qi, Jingjing Ren, Shuzhu Cao, Yongjian Li, Keshuang Li, Mengying Du, Shilei Zhang, Lin Yang, Yongjie Wang, Yayin Qi

**Affiliations:** 1 College of Animal Science and Technology, Shihezi University, Shihezi 832003, China; 2 Department of Animal Science, College of Agriculture and Environmental Science, North Carolina Agricultural and Technical State University, Greensboro, NC 27411, USA

**Keywords:** neonatal meningitis-associated *E. coli* (NMEC), whole-genome sequencing (WGS), multidrug resistance (MDR), hybrid pathotype, virulence factors, extraintestinal pathogenic *E. coli* (ExPEC), calf meningitis

## Abstract

Neonatal meningitis-associated *Escherichia coli* (NMEC) is a formidable pathogen in veterinary medicine. The emergence of atypical, multidrug-resistant (MDR) variants complicates disease control. An *Escherichia coli* (*E. coli*) strain was isolated from the brain tissue of a deceased calf with acute meningitis. Comprehensive characterizations were performed, including whole-genome sequencing (WGS), multi-locus sequence typing (MLST), antimicrobial susceptibility testing (AST), murine pathogenicity assays, and RT-qPCR evaluation of neuroinflammatory cytokines. Results: The isolate (O18ab:H14) was identified as a capsule-deficient NMEC strain belonging to phylogroup A and sequence type ST1434. WGS showed that the genome size of this strain is 5.1 Mb, containing 73 strictly defined antimicrobial resistance genes and 202 virulence factors. These may be involved in the compensatory mechanism for capsule deficiency, and further functional verification is required. Phenotypically, it exhibited a robust MDR profile. In the murine model, the strain demonstrated high lethality, and induced severe multi-organ lesions characteristic of both meningitis and systemic sepsis. While intraperitoneal injection bypasses natural colonization routes, the brain-specific bacterial persistence and neuronal pathology imply neurotropic potential. Furthermore, RT-qPCR confirmed a severe neuroinflammatory response, marked by the significant upregulation of IL-1β, IL-6, and TNF-α in the infected brains. This study characterizes a novel, highly virulent, and MDR capsule-deficient NMEC/SEPEC hybrid strain. The findings emphasize the urgent need for continuous genomic surveillance of atypical *E. coli* pathotypes in livestock.

## Simple Summary

Severe brain infections caused by the bacterium *Escherichia coli* result in high death rates among newborn calves, creating major economic losses for the farming industry. Typically, these harmful bacteria rely on a protective outer shell, known as a capsule, to hide from the animal’s immune system. This study aimed to investigate a unique bacterial strain isolated from a calf that died from severe brain inflammation. Surprisingly, genetic analysis showed that this specific bacterium lacks the traditional protective capsule. Instead, it is equipped with an unusually high number of genetic traits that make it resistant to multiple medicines and highly capable of causing disease. When tested in an animal model, this unusual strain proved to be highly deadly, causing severe inflammation in the brain and damaging multiple internal organs. These findings demonstrate that bacteria can develop unexpected alternative strategies to remain dangerous, even without typical protective features. For society, this research emphasizes the urgent need to continuously monitor evolving, medicine-resistant bacterial strains on livestock farms. Such vigilance is essential to protecting animal welfare, safeguarding the agricultural food supply, and preventing the spread of difficult-to-treat infections.

## 1. Introduction

ExPEC represents a highly diverse and lethal group of pathogens capable of infecting both humans and animals, thereby posing a significant threat to global public health and animal welfare [1]. Unlike commensal *E. coli* residing in the intestinal tract, ExPEC strains have acquired specialized virulence factors that facilitate their invasion, survival, and dissemination within extraintestinal host niches. These versatile pathogens are responsible for a wide spectrum of severe systemic diseases, including neonatal meningitis, urinary tract infections (UTIs), pneumonia, osteomyelitis, and profound sepsis in young animals [2]. The remarkable adaptability and pathogenicity of ExPEC are largely driven by a complex arsenal of virulence factors, predominantly including adhesins (which mediate host cell attachment), toxins (which induce tissue damage), invasins, outer membrane proteins (OMPs), and highly efficient iron-acquisition systems that ensure bacterial survival in iron-depleted host environments [3].

Within the ExPEC group, three major sub-pathotypes are widely recognized: neonatal meningitis-associated *E. coli* (NMEC), uropathogenic *E. coli* (UPEC), and avian pathogenic *E. coli* (APEC). Among these, NMEC is particularly devastating as it specifically targets the central nervous system (CNS) [4]. In both human infants and neonatal animals, NMEC infection is a severe, life-threatening condition [5]. Survivors frequently suffer from permanent and debilitating neurological sequelae, such as encephalitis, brain abscesses, ventriculitis, cerebral atrophy, hydrocephalus, hearing loss, developmental defects, and epilepsy [6]. In veterinary medicine, NMEC is recognized as the most common Gram-negative bacterium causing meningitis in calves, with the highest susceptibility observed in neonates aged 3 to 8 days [7]. The pathogenesis of bovine NMEC typically begins with the entry of the pathogen through the respiratory tract, gastrointestinal tract, or an unhealed umbilical cord [8]. Following initial colonization, the bacteria breach the epithelial barriers, enter the bloodstream to induce severe bacteremia (colisepticemia), and eventually cross the blood–brain barrier (BBB) to cause fatal meningitis [9]. Clinically, infected calves exhibit acute high fever, severe lethargy, ataxia, blindness, loss of the suckling reflex, and opisthotonos, rapidly progressing to death [10]. These sudden outbreaks not only compromise animal welfare but also inflict devastating economic losses on the cattle breeding industry [11]. In recent years, the clinical management of NMEC has become increasingly challenging due to the emergence of MDR strains and variant pathotypes [12]. In early December 2024, a severe disease outbreak occurred at a large-scale cattle farm in Xinjiang, China. The affected neonatal calves presented with acute clinical signs, including high fever, loss of appetite, severe depression, diarrhea, and progressive emaciation, culminating in rapid mortality [13]. Subsequent necropsies consistently revealed severe meningeal hemorrhage and cerebral congestion, which are highly indicative of acute bacterial meningitis [14].

To elucidate the etiology of this outbreak and comprehend the underlying pathogenic mechanisms, a 6-day-old deceased calf was selected as the primary subject for this study. Brain tissue samples were aseptically collected for the isolation and identification of the causative bacterial pathogen using standard microbiological methods. To thoroughly characterize the isolate, we conducted a comprehensive systematic analysis encompassing MLST, AST, virulence gene profiling, and in vivo pathogenicity assays. Furthermore, WGS was employed to map the complete genetic landscape of the strain. By systematically investigating its biological characteristics and pathogenic mechanisms at the genomic level, this study aims to provide a robust theoretical foundation for understanding NMEC infections in calves and to offer critical insights for the prevention and control of this lethal disease in the Xinjiang region.

## 2. Materials and Methods

### 2.1. Ethical Statement

All animal experiments were conducted in strict accordance with the recommendations in the Guide for the Care and Use of Laboratory Animals. The in vivo pathogenicity study protocol was reviewed and approved by the Animal Care and Ethics Committee of Shihezi University (Approval No. A2026-220).

### 2.2. Sample Collection and Bacterial Isolation

In a large cattle farm in Xinjiang, China, 6 calves showed neurological symptoms, with a mortality rate of 50%. Necropsies all showed meningeal lesions. A typical acutely ill individual (6 days old) was selected. Due to the significant neurological symptoms and no antibacterial treatment, an necropsy was immediately performed after death. The brain tissue samples were collected under strict aseptic conditions and temporarily preserved on ice for immediate transport to the laboratory. For bacterial isolation, the surface of the infected brain tissue was sterilized, and a small portion was excised in a biosafety cabinet. The tissue was homogenized and inoculated into Luria–Bertani (LB) broth (Hope Bio-Technology Co., Ltd., Qingdao, China) and incubated at 37 °C for 18–24 h to enrich the bacterial population. Subsequently, a loopful of the enriched culture was streaked onto LB solid agar plates and incubated at 37 °C for 12 h. Single colonies were carefully picked and subcultured in liquid LB broth for another 24 h. Preliminary morphological characterization and purity verification were performed using Gram staining under a light microscope [15]. Serial quadrant streaking was performed on agar plates for three consecutive generations to ensure isolate purity. Uniform colony morphology and monomicrobial Gram stain results (Gram-negative rods) confirmed the establishment of an axenic culture. Finally, the purified isolate was cultured on Eosin Methylene Blue (EMB) agar and MacConkey (MAC) agar to observe the specific colony morphology characteristic of *E. coli*. Biochemical analysis was performed according to the standard protocol for the identification of Enterobacteriaceae. The isolates were inoculated into the biochemical identification tube media of MR-VP, Glucose, Sucrose, Lactose, and Mannitol (Hope Bio-Technology). After incubation at 37 °C for 24 h, the negative and positive results were recorded.

### 2.3. Molecular Identification, WGS and Phylogenetic Analysis

The genomic DNA of the purified isolate was extracted using a Bacterial Genomic DNA Extraction Kit (Vazyme Biotech Co., Ltd., Nanjing, China) according to the manufacturer’s instructions. Molecular identification was performed via Polymerase Chain Reaction (PCR) using primers specific for *E. coli* species-discriminatory genes (gapA) and universal bacterial 16S rRNA region (Table 1). The 20 μL PCR mixture contained 10 μL of 2× San Taq PCR Mix (Vazyme), 1 μL of each forward and reverse primer, 2 μL of template DNA, and 6 μL of nuclease-free water. The thermocycling conditions consisted of an initial denaturation at 95 °C for 3 min; 30 cycles of denaturation at 95 °C for 15 s, annealing at 60 °C for 15 s, and extension at 72 °C for 30 s; followed by a final extension at 72 °C for 5 min. The PCR products were separated on a 1% agarose gel to confirm the specificity of the amplicon size. Subsequently, the 16S rRNA PCR products were sent to Xinjiang Youkang Biotechnology Co., Ltd. (Urumqi, China) for bidirectional Sanger sequencing using 16S rRNA region amplification primers (forward and reverse). For WGS, the genomic DNA was submitted to Shanghai Lingen Biotechnology Co., Ltd. (Shanghai, China). The third-generation single-molecule sequencing technology (PacBio RS II/Sequel) was used to obtain long reads, and the second-generation high-throughput sequencing (Illumina) was combined to obtain short reads. Trimmomatic (v0.36) was used to filter the raw data with the parameters: ILLUMINACLIP:adapters.fa:2:30:10 SLIDINGWINDOW:4:15 MINLEN:75 to obtain high-quality clean data [16]. Hybrid assembly of second-generation and third-generation sequencing data through the Unicycler pipeline to optimize assembly continuity [17]. The NCBI ab initio prediction was combined with the GeneMark optimization model and verified by BLASTp (Diamond: 0.9.22.123) alignment against the NR database [18]. Gene function annotation was performed through databases such as Gene Ontology (GO) [19], Cluster of Orthologous Groups (COG) [20], Kyoto Encyclopedia of Genes and Genomes (KEGG) [21], Virulence Factors Database (VFDB) [22] and Comprehensive Antibiotic Resistance Database (CARD) [23]. The raw image data obtained from sequencing were converted into sequence data through base calling, and the results were stored in the FASTQ file format. To elucidate the evolutionary relationship of the isolate, homologous sequence alignment was performed, and a phylogenetic tree was constructed using MEGA 11.0 software [24].

### 2.4. Growth Curve Determination

To evaluate the growth kinetics of the isolate, a freshly cultured bacterial suspension was inoculated into 5 mL of Lysogeny Broth (LB) medium (Hope Bio-Technology) at a ratio of 1:50. The culture was incubated at 37 °C with continuous shaking. The optical density at 600 nm (OD_600_) was measured every 2 h for a total duration of 24 h. The assay was performed in triplicate (three independent parallel biological replicates), and a growth curve was plotted based on the mean OD_600_ values [25].

### 2.5. Serotyping, Phylogrouping, and MLST

The assembled WGS data were uploaded to the SerotypeFinder web server (https://cge.food.dtu.dk/services/SerotypeFinder/, accessed on 15 May 2025) [26] to predict the *E. coli* serotype. To determine the phylogenetic group, a triplex PCR assay targeting the *chuA*, *yjaA*, and *TspE4.C2* genes was conducted. The 20 μL reaction mixture contained: 10 μL of 2× Taq PCR Master Mix, 1 μL of each primer, and 6 μL nuclease-free water. The phylogroups were classified based on the amplification patterns of *chuA*, *yjaA*, and *TspE4.C2* genes according to the standard criteria established by Clermont et al. (2013) [27]: Group A: *chuA* negative, *yjaA* negative, Group B1: *chuA* negative, *yjaA* negative, *TspE4.C2* positive, Group B2: *chuA* positive, *yjaA* positive, Group D: *chuA* positive, *yjaA* negative, Group E: *chuA* negative, *yjaA* positive (if detected). This empirical result was further validated in silico using the ClermontTyping online tool (http://clermontyping.iame-research.center, accessed on 23 May 2025) with the WGS data. For MLST, seven standard housekeeping genes (*adk*, *fumC*, *gyrB*, *icd*, *mdh*, *purA*, and *recA*) were amplified via PCR using primers and cycling conditions specified by the *E. coli* MLST scheme [28]. After purification, the PCR products were subjected to bidirectional Sanger sequencing using the original amplification primers by Xinjiang Youkang Biotechnology Co., Ltd. The allelic profiles and corresponding Sequence Type (ST) were determined by querying the *E. coli* MLST database (https://pubmlst.org/databases/, accessed on 16 June 2025) [29].

### 2.6. Virulence and Antimicrobial Resistance Gene Annotation

To systematically explore the pathogenic potential and resistance mechanisms, the whole-genome data were screened against the Virulence Factor Database (VFDB, https://www.google.com/search?q=http://www.mgc.ac.cn/VFs, accessed on 9 November 2025) and the Comprehensive Antibiotic Resistance Database (CARD, https://card.mcmaster.ca/, accessed on 13 November 2025). Genes associated with virulence factors and antimicrobial resistance were structurally annotated, enabling the prediction of related regulatory pathways and potential pathological threats.

### 2.7. Antimicrobial Susceptibility Testing

Based on the CARD genomic predictions, the phenotypic antimicrobial susceptibility of the isolate was evaluated using the Kirby-Bauer (K-B) disk diffusion method. A total of 15 commercial antibiotic disks (Microbial Reagent Co., Ltd., Hangzhou, China) were tested, including gentamicin (R: ≤12 mm, I: 13–14 mm, S: ≥15 mm), ciprofloxacin (R: ≤15 mm, I: 16–20 mm, S: ≥21 mm), polymyxin (R: ≤11 mm, I: 12–14 mm, S: ≥15 mm), tetracycline (R: ≤11 mm, I: 12–14 mm, S: ≥15 mm), rifampicin (R: ≤16 mm, I: 17–19 mm, S: ≥20 mm), amoxicillin (R: ≤13 mm, I: 14–16 mm, S: ≥17 mm),streptomycin (R: ≤11 mm, I: 12–14 mm, S: ≥15 mm), enrofloxacin (R: ≤15 mm, I: 16–20 mm, S: ≥21 mm),ofloxacin (R: ≤12 mm, I: 13–15 mm, S: ≥16 mm), and cefepime (R: ≤23 mm, I: 24–26 mm, S: ≥27 mm). Briefly, the bacterial suspension (cultivate to the logarithmic phase which was detected by a microplate reader) was uniformly spread onto LB agar plates (100 μL per plate). The antibiotic disks were then aseptically placed on the agar surface. After a 16 h incubation at 37 °C, the diameters of the inhibition zones were measured and interpreted according to the Clinical and Laboratory Standards Institute (CLSI) guidelines to determine the susceptibility profile [30].

### 2.8. Detection of Virulence-Associated Genes

Guided by the VFDB prediction results, four classic virulence genes highly associated with NMEC pathogenicity (*ompA*, *fimH*, *nlpI*, and *malX*) were selected for molecular validation. Virulence-associated genes were detected by PCR using primers listed in Table 2. The 20 μL reaction mixture contained: 10 μL 2× Taq Master Mix, 1 μL each primer, 2 μL DNA, and 6 μL nuclease-free water. Thermal cycling conditions followed Section 2.3 [31]. Specific primers for these genes were designed based on sequences available in the NCBI database and synthesized by Xinjiang Youkang Biotechnology. The presence of these genes in the isolate was confirmed using conventional PCR.

### 2.9. In Vivo Pathogenicity and Histopathology Evaluation

To determine the median lethal dose (LD_50_) and evaluate in vivo pathogenicity, 36 Specific-Pathogen-Free (SPF) Kunming mice were randomly divided into six groups (*n* = 6 per group). The bacterial concentration was determined via the plate counting method, and the culture was serially diluted in sterile PBS to five concentrations: 1 × 10^9^, 1 × 10^8^, 1 × 10^7^, 1 × 10^6^, and 1 × 10^5^ CFU/mL. Mice in Groups 1 to 5 were intraperitoneally (i.p.) injected with 0.2 mL of the respective bacterial dilutions, while Group 6 (negative control) received 0.2 mL of sterile PBS [32].

The mice were closely monitored for 7 days to record clinical signs, feed/water intake, and mortality rates. Deceased mice were immediately subjected to necropsy. Key organs, including the brain, liver, lungs, and spleen, were harvested, examined for gross pathological lesions, and fixed in 4% paraformaldehyde. The fixed tissues were subsequently sent to Wuhan Servicebio Technology Co., Ltd. (Wuhan, China) for paraffin embedding, sectioning, and Hematoxylin-Eosin (H&E) staining to observe histopathological alterations under a light microscope.

### 2.10. Quantitative Real-Time PCR (RT-qPCR) for Immune Factors in Brain Tissue

To assess the neuroinflammatory response induced by the infection, the mRNA expression levels of key cytokines (IL-1β, IL-6, TNF-α, and IFN-γ) in the brain tissues of deceased mice (infection group) and PBS-treated mice (control group) were quantified. Briefly, the collected brain tissues were rapidly ground into a fine powder using a tissue homogenizer. Total RNA was extracted using the TransZol Up Plus RNA Kit (TransGen Biotech, Beijing, China). RNA concentration and purity were measured, and the samples were stored at −80 °C. Reverse transcription was performed using the TransScript^®^ One-Step gDNA Removal and cDNA Synthesis SuperMix (TransGen Biotech). A 20 μL reaction mixture (containing 1 μL Total RNA, 4 μL 5× All-in-One Reaction Mix, 1 μL TransScript^®^ Enzyme Mix, and 14 μL RNase-free water) was incubated at 50 °C for 5 min, followed by enzyme inactivation at 85 °C for 2 min.

RT-qPCR was conducted using the Taq Pro Universal SYBR qPCR Master Mix on a fluorescence quantitative PCR system. The mouse β-actin gene was used as the endogenous reference control. The 10 μL PCR system comprised 5 μL of SYBR qPCR Master Mix, 0.2 μL of each forward and reverse primer, 1 μL of cDNA template, and 3.6 μL of *dd*H_2_O. The primers used are shown in Table 3. The thermal cycling program consisted of an initial pre-denaturation at 95 °C for 30 s, followed by 40 cycles of denaturation at 95 °C for 3 s and annealing/extension at 60 °C for 30 s. Independent sample *t*-test was used to test the significance of differences. All samples were analyzed in biological and technical triplicates. The reference gene selected is β-actin. The data in the figures were presented as mean ± standard deviation (mean ± SD). The relative expression levels of the target genes were calculated using the 2^−△△Ct^ method, and the results were visualized using OriginPro 2021 software.

## 3. Results

### 3.1. Isolation, Identification, and Biological Characteristics of the Pathogen

**Figure 1:**
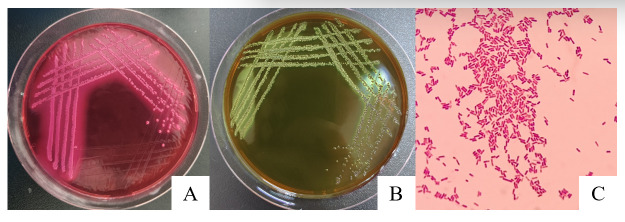
Bacteria grown on MAC agar (**A**), Bacteria grown on EMB agar (**B**), Gram staining of isolated bacteria (**C**).

Bacterial isolation from the brain tissue of the deceased calf yielded presumptive *E. coli* colonies on selective media. Specifically, the isolate formed characteristic pink colonies with distinct precipitation halos on MAC agar (Figure 1A) and distinctive colonies with a blue-green metallic sheen on EMB agar (Figure 1B). Gram staining was performed on the bacteria of each culture. Microscopic examination following Gram staining revealed Gram-negative, rod-shaped bacteria occurring either singly or in pairs (Figure 1C), consistent with typical *E. coli* morphology.

Subsequent biochemical profiling confirmed this preliminary identification. The isolate tested positive for glucose, sucrose, lactose, and mannitol fermentation, while yielding a negative result in the Methyl Red-Voges Proskauer (MR-VP) test (Table 4). These biochemical traits firmly align with the standard profile of *E. coli*.

**Figure 2:**
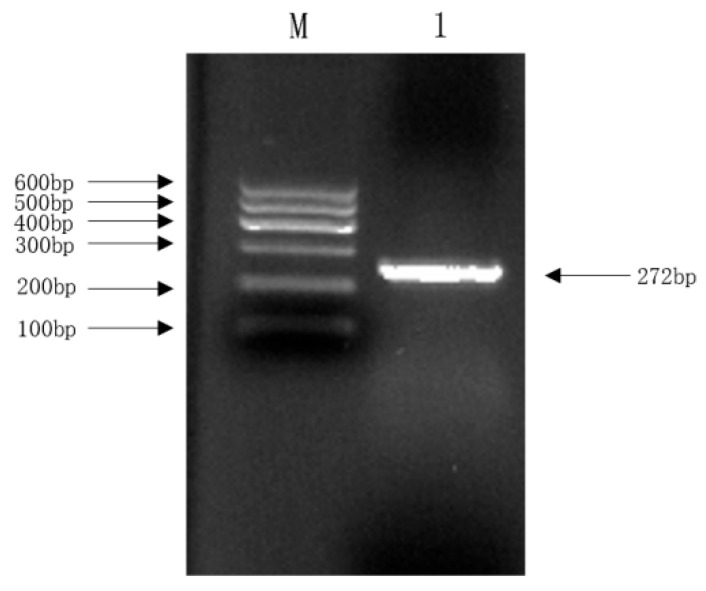
PCR amplification of *E. coli* species-discriminatory genes. Note: M. DL600 DNA Marker; 1. *E. coli* species-discriminatory genes (gapA).

**Figure 3:**
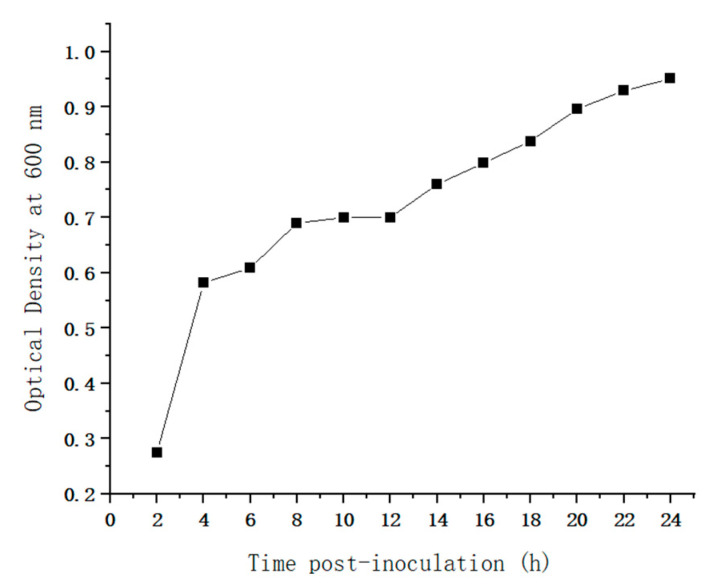
Growth curve of bacteria isolate in LB medium at 37 °C.

Molecular identification was performed using PCR targeting the 16S rRNA region. The resulting amplicon matched the expected size, and subsequent BLAST analysis of the sequenced 16S rRNA region showed a 99.41% homology with established *E. coli* sequences in the NCBI database, definitively confirming the isolate as *E. coli* (Figure 2). To assess the proliferation capacity of the isolate, a 24 h growth curve was established by monitoring the optical density at 600 nm (OD_600_). As depicted in Figure 3, the bacterial population entered the logarithmic (exponential) growth phase approximately 2 h post-inoculation, marked by a rapid and significant increase in OD_600_. After 20 h of continuous culture, the growth rate decelerated, transitioning into the stationary phase (Figure 3).

### 3.2. Genomic Typing and Phylogenetic Analysis

Whole-genome sequence (WGS) data were utilized for comprehensive molecular typing. Serotype prediction using the SerotypeFinder database identified the isolate as serotype O18ab:H14. For phylogenetic grouping, triplex PCR amplification yielded no specific bands for *chuA*, *yjaA*, or *TspE4.C2*, classifying the isolate into Phylogroup A. This finding was further validated in silico using the ClermontTyping tool based on the assembled genome. MLST based on seven standard housekeeping genes (*adk*, *fumC*, *gyrB*, *icd*, *mdh*, *purA*, and *recA*) identified the strain as sequence type ST1434. Specifically, the allele numbers of *fumC*, *gyrB*, *icd*, *mdh*, *purA*, and *recA* were assigned as 10, 11, 5, 8, 7, 8, and 6, respectively. The ST of the isolated was ST1434.

**Figure 4:**
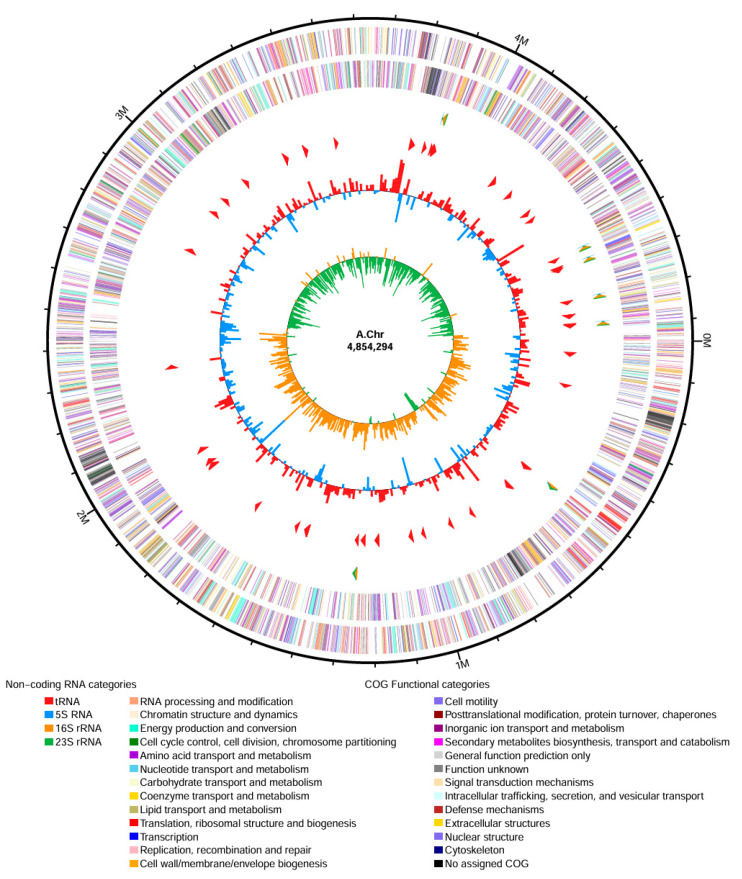
Circular genome map of strain. Note: From the outside to the inside, Ring 1: size of the genome; Ring 2: Coding sequences (CDS) on forward strand; Ring 3: CDS on reverse strand; Ring 4: rRNA/tRNA locations; Ring 5: GC content; Ring 6: GC skew.

**Figure 5:**
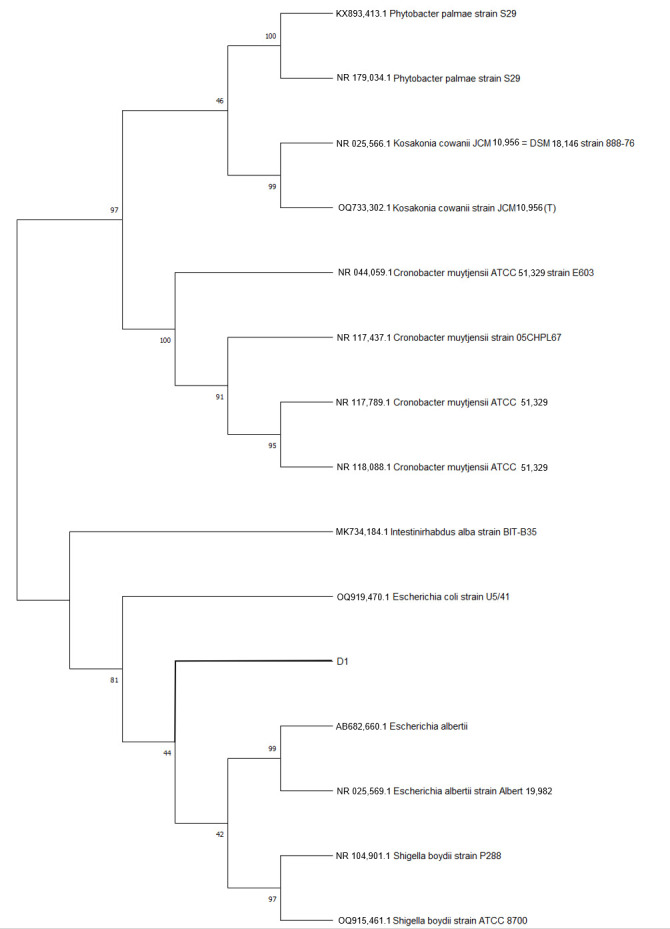
Phylogenetic tree based on 16S rRNA region sequences. Isolate in this study is shown in the branch in bold-D1.

After filtering the chromosomal genome of the isolated, the number of effective reads was 275,112, with a total length of 1,374,934,169 bp, an average length of 4997 bp, and a maximum length of 82,918 bp. After gene assembly, the total length was 5,046,008 bp, the G+C content was 50.98%, and there were no unknown bases. The N50 is 4,854,294 bp. The complete genome of the isolated strain comprises a single circular chromosome of 5,124,195 bp. Gene prediction identified a total of 4695 genes, consisting of 4586 coding sequences (CDS) and 109 non-coding RNAs (accounting for 2.32% of the total genome). The non-coding elements include 87 tRNAs (1.85%) and 22 rRNAs (0.47%). Furthermore, the genome harbors two CRISPR arrays (both with 29 repeat sequences) and ten predicted prophage regions of varying lengths. A circular genomic map was constructed to visualize these genomic features (Figure 4). Based on the genomic sequence, a Neighbor-Joining phylogenetic tree was constructed using MEGA 11.0 (with 1000 bootstrap replicates). The evolutionary tree demonstrated that the isolate clustered tightly within the *E. coli* clade, sharing high sequence similarity with reference strains AB682660.1, NR025569.1, NR104901.1, and OQ915461.1 (Figure 5).

### 3.3. Antimicrobial Resistance Profile and Resistance Genes

**Figure 6:**
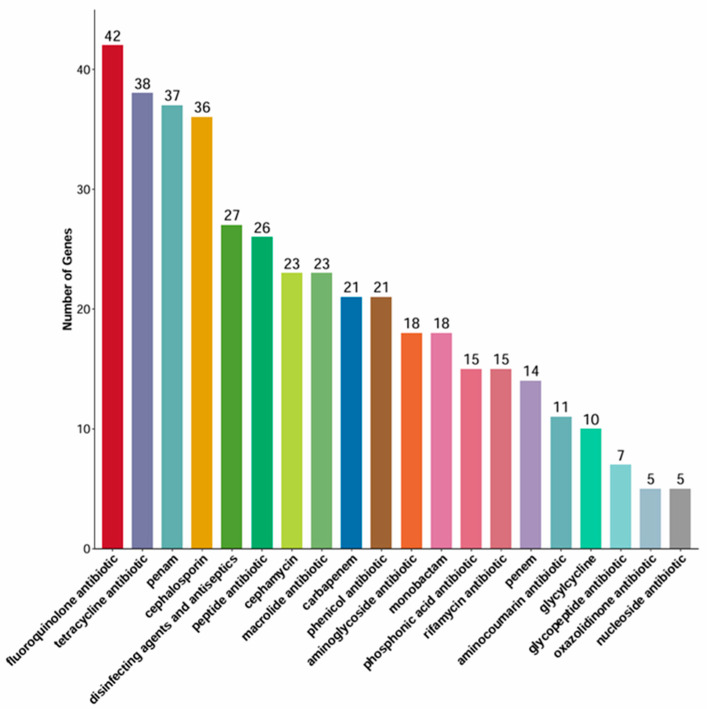
CARD-based annotation of resistance genes identified in the whole-genome sequence of the isolate categorized by mechanism.

Genomic screening against the Comprehensive Antibiotic Resistance Database (CARD) identified a staggering 161 resistance-associated genes (Figure 6, Supplement Table S1). These genes encompass seven distinct mechanisms of resistance: antibiotic target alteration (60 genes, e.g., *PmrF*, *bacA*), antibiotic efflux (93 genes, e.g., *cpxA*, *acrB*), antibiotic inactivation (4 genes, e.g., *ampR*, *cmlv*), antibiotic target protection (1 genes, *optrA*), reduced permeability to antibiotics (13 genes, e.g., *MdtQ*, *mipA*), resistance by absence (4 genes, e.g., *LamB*, *PhoP*), and antibiotic target replacement (2 genes, e.g., *rpoB*, *dfrA3*). Notably, several genes exhibited dual functions, such as conferring both efflux and target alteration (11 genes, e.g., *soxR*, *AcrR*). Applying stringent criteria (coverage ≥80%, consistency ≥90%, and clear resistance mechanism), we defined 73 genes as strict ARGs. Clinically relevant ARGs included: (1) core resistance genes directly mediating antibiotic inactivation or target alterations (e.g., *gyrB*, *folP*, *rpoB*, *gyrA*, *parC*, *parE*, *CTX-M-14*, *PBP3*, *uhpA*, *uhpT*) and efflux pumps triggering multidrug resistance (e.g., *mdfA*, *emrE*); (2) indirect regulatory systems including efflux complexes (AcrAB-TolC regulators like *AcrR*, *MarR*), mdt series genes (*mdtG*, *mdtM*), transcriptional factors (*marA*, *soxR/S*, *evgA/S*), lipid A modification (*PmrF*), and membrane structure genes (*mlaF*, *ugd*); and (3) specialized mechanisms such as *fabI/fabG*, *nfsA*, and *EF-Tu*.

The in vitro antimicrobial susceptibility test (AST) evaluating 15 common antibiotics largely corroborated the genomic predictions. The strain exhibited 100% resistance (R) to amoxicillin. It also showed resistance to gentamicin, tetracycline, ciprofloxacin, rifampicin and cefepime. The strain demonstrated intermediate susceptibility (I) to neomycin, polymyxin, streptomycin, enrofloxacin, ofloxacin, puromycin, and cefepime, while showing no susceptibility (S) to any of the tested antibiotics (Table 5). The resistance phenotypes for β-lactams, macrolides, aminoglycosides, and tetracyclines strongly aligned with the identified resistance genes. Interestingly, while the genome harbored genes conferring resistance to carbapenems and cephalosporins, the phenotypic assay indicated that active clinical resistance to these classes has not yet fully developed. The experiment follows the Interpretation per CLSI M100 2024 [30] standard and only includes antibiotics with interpretation criteria for Enterobacteriaceae.

### 3.4. Virulence Factors and In Vivo Pathogenicity

#### 3.4.1. Virulence Factors

**Figure 7:**
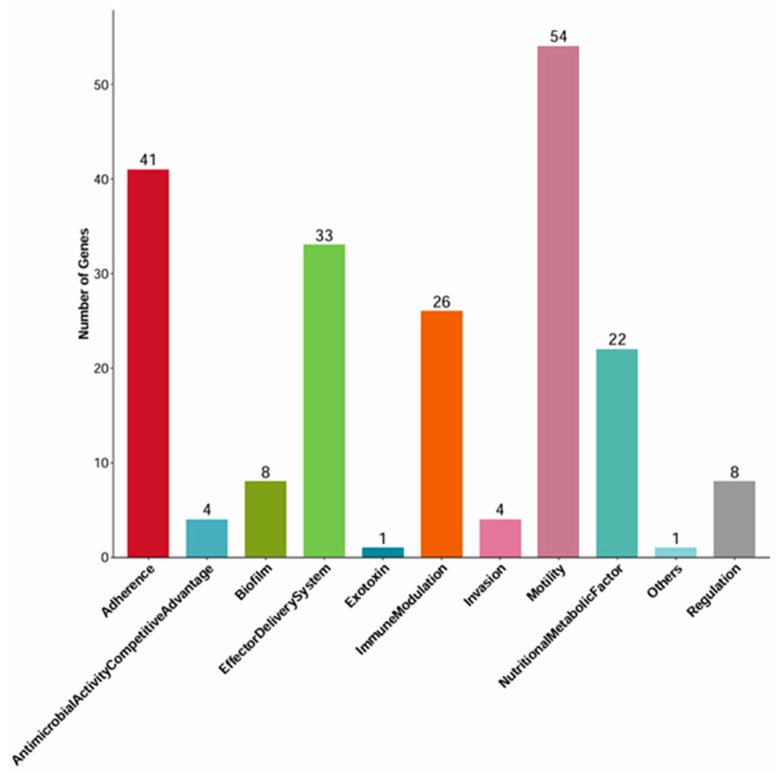
Distribution of virulence factor genes identified in the whole-genome sequence of the isolate categorized by factor functional categories.

Analysis using the Virulence Factor Database (VFDB) annotated 202 genes corresponding to 180 distinct virulence factors (VFs). These VFs were categorized into 11 functional groups, predominantly involving motility (30%, 54 genes including *fliC*, *flk*, *fliR*, etc.) and effector delivery systems (25%, 33 genes). Only one gene (*hlyE*/*clyA*) was associated with exotoxin function (Figure 7). A detailed list and the total number of virulence factors are provided in the Supplementary Materials (Supplement Table S2). To confirm the expression of specific virulence genes predicted by WGS, we selected four key NMEC-related pathogenic genes for PCR: *ompA* (outer membrane protein), *nlpI* (lipoprotein), *fimH* (pilus adhesin), and *malX* (pathogenicity island marker). The positive amplification results supported the genome annotation.

#### 3.4.2. In Vivo Pathogenicity

**Figure 8:**
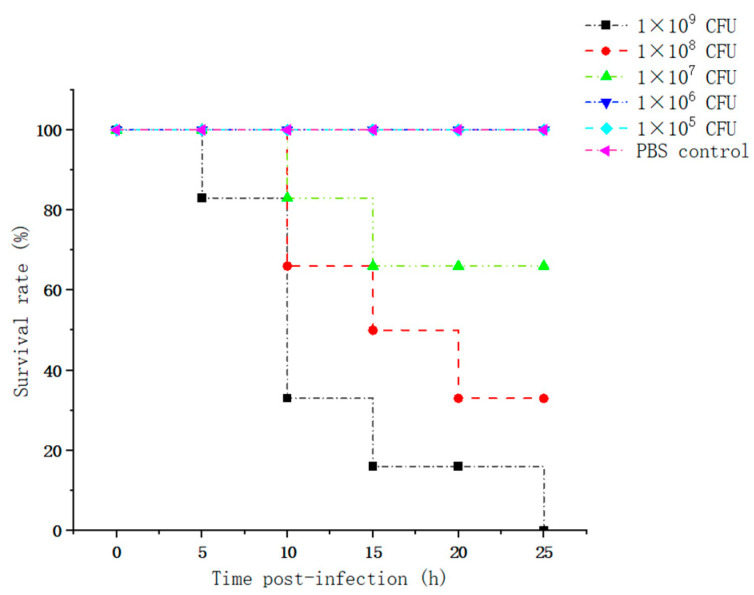
Survival curves of mice infected with different doses of the bacteria isolate.

**Figure 9:**
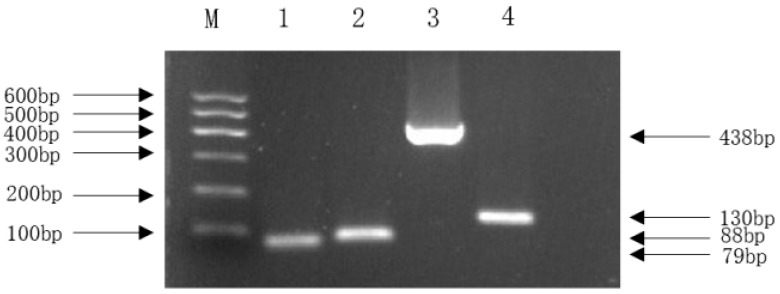
PCR amplification of virulence genes of isolated bacteria. Note: M. DL600 DNA Marker; 1. *ompA* (79 bp); 2. *nlpI* (88 bp); 3. *fimH* (438 bp); 4. *malX* (130 bp).

**Figure 10:**
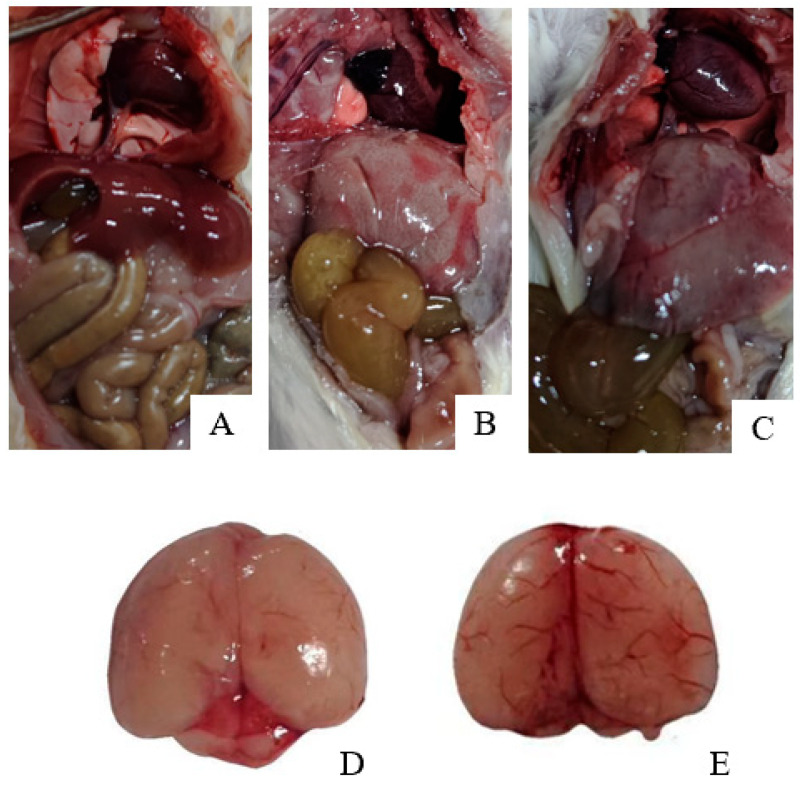
Gross anatomical observations in control and challenged mice. (**A**) Control group (PBS)—systemic view; (**B**,**C**) Challenge group (1 × 10^9^ CFU)—systemic view; (**D**) Control group (PBS)—Brain tissue; (**E**) Challenge group (1 × 10^9^ CFU)—Brain tissue.

**Figure 11:**
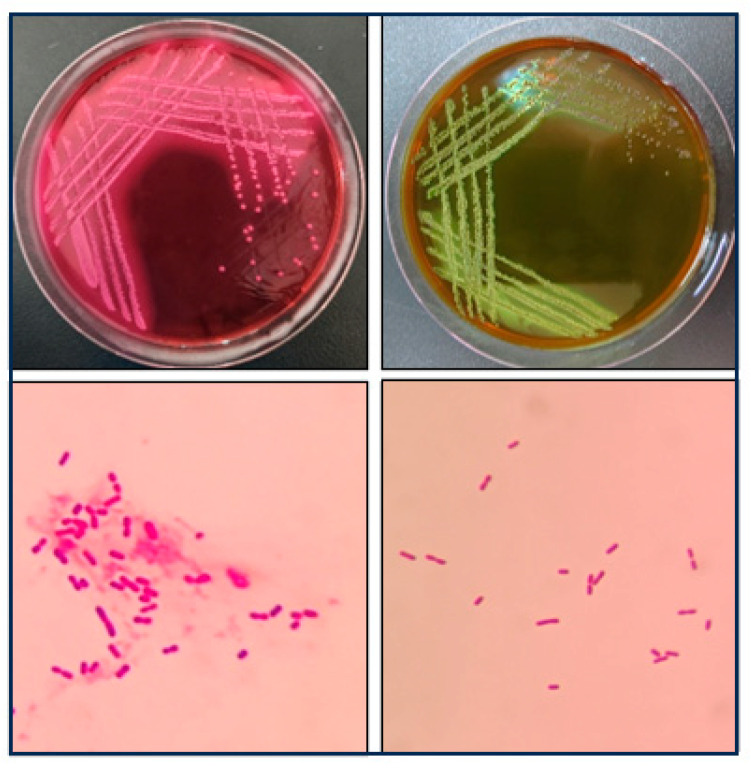
Morphology of single colonies formed by bacteria isolated from the brain tissue of mice after toxin challenge on MAC agar and EMB agar and the results of Gram staining.

**Figure 12:**
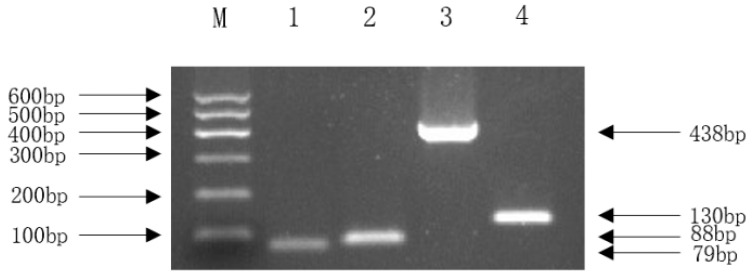
PCR amplification of bacteria isolated from the brain tissue of mice after bacteria isolate challenge. Note: M. DL600 DNA Marker; 1. *ompA* (79 bp); 2. *nlpI* (88 bp); 3. *fimH* (438 bp); 4. *malX* (130 bp).

**Figure 13:**
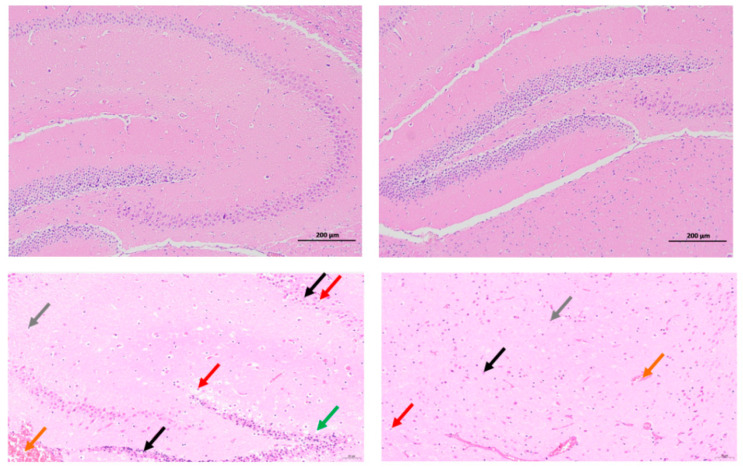
Histopathological alterations in murine brain tissue following NMEC infection (H&E staining, 100×). (**Top**: Control group; **Bottom**: Infected group [1 × 10^9^ CFU]) Meningeal vascular congestion (orange arrows); Necrosis with nuclear fragmentation (black arrows); Cytoplasmic edema (red arrows); Glial response: Mild proliferation (gray arrows); Interstitial lesions: Microvacuolation (green arrows).

**Figure 14:**
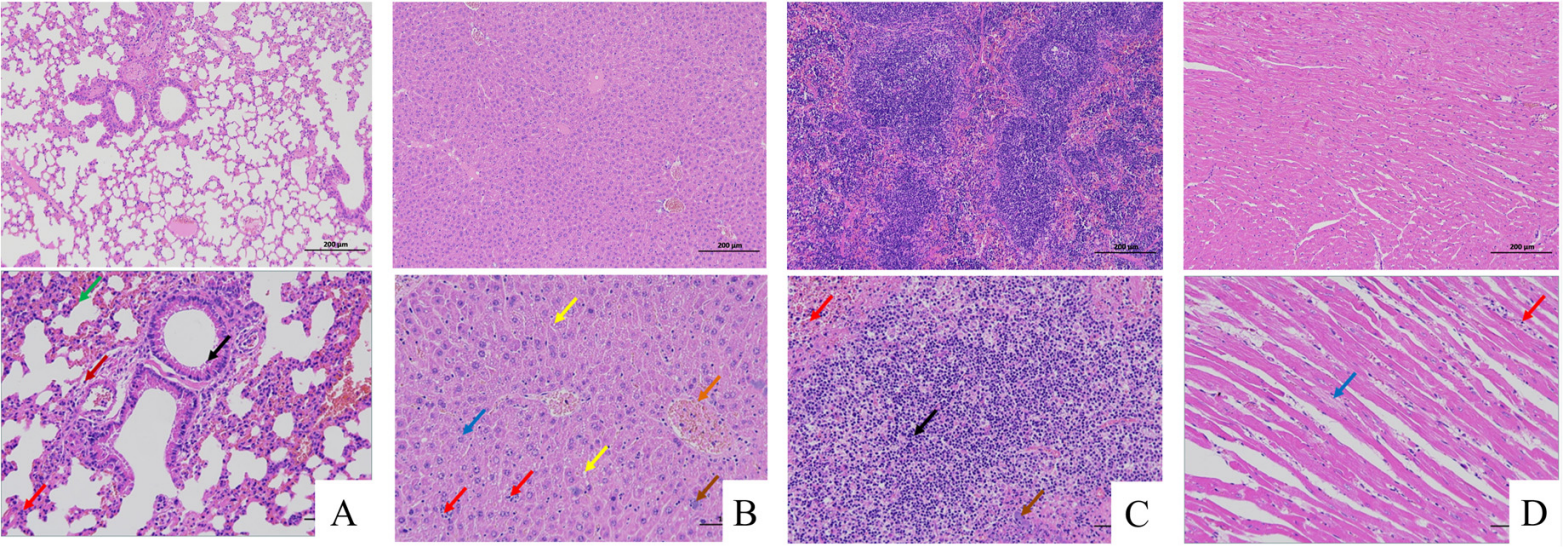
Systemic histopathology in NMEC-infected mice (H&E staining, 100×). (**Top**: Control group; **Bottom**: Infected group [1 × 10^9^ CFU]) (**A**) Lung, Granulocyte infiltration (red arrow); Alveolar hemorrhage (green arrow); Eosinophilic substances (black arrow); Lymphocyte infiltration (dark red arrow); (**B**) Liver, Fatty degeneration (yellow arrow); Hepatocyte edema (blue arrow); Focal infiltration (red arrow); Basophilic masses (brown arrow);Vascular congestion (orange arrow); (**C**) Spleen, Lymphocyte necrosis (black arrow); Granulocyte infiltration (red arrow); Basophilic masses (brown arrow);. (**D**) Heart, Loose cytoplasm of cardiomyocytes (blue arrow); Lymphocyte infiltration (red arrow).

The in vivo pathogenicity of the isolate was evaluated using an intraperitoneal murine challenge model. Mice in the high-dose infection groups (1 × 10^7^ CFU–1 × 10^9^ CFU) exhibited acute onset of disease within 5 h post-inoculation, displaying severe clinical signs including complete anorexia, lethargy, huddling due to chills, ruffled fur, severe depression, increased ocular discharge, and diarrhea. Mortality was highly dose-dependent. Mice infected with 1 × 10^9^ CFU/mL began dying at 5 h, reaching a 100% mortality rate (6/6). Those infected with 1 × 10^8^ and 1 × 10^7^ CFU/mL began dying at 10 h, with mortality rates of 66% (4/6) and 33% (2/6), respectively. Mice in the lower dose groups (1 × 10^6^ and 1 × 10^5^ CFU/mL) developed mild transient symptoms but fully recovered after 24 h with no mortality. The negative control group remained healthy throughout the observation period (Figure 8). To confirm the isolate’s ability to cross the BBB, brain tissues from the deceased mice were aseptically harvested. Bacterial recovery assays successfully cultured colonies exhibiting the characteristic *E. coli* morphology on MAC and EMB agar. Subsequent PCR detection from these brain isolates confirmed the presence of the exact virulence gene profile (*ompA*, *nlpI*, *fimH*, *malX*) as the original challenge isolate (Figure 9), definitively demonstrating the pathogen’s capacity to penetrate the BBB and colonize the central nervous system. Gross necropsy of the deceased mice revealed multi-organ involvement typical of acute sepsis and meningitis. Pathological findings included cerebral surface hemorrhage and swelling; severe hepatomegaly with massive petechiae and ecchymoses; varying degrees of hemorrhage and enlargement in the lungs, spleen, and myocardium; and flaccid, edematous intestines containing foul-smelling yellowish-brown fluid (Figure 10). Mice were artificially infected by intraperitoneal injection of bacterial culture. Immediately after death, mice were necropsed, and brain tissue was aseptically collected. The tissue was then inoculated into BHI liquid medium and cultured at 37 °C for 12 h. After culture, the culture was inoculated into EMB agar and MAC agar differential media. Colonies with a blue-green metallic sheen and a pinkish-red color with a distinct precipitate ring were observed. Microscopic examination revealed long, rod-shaped, blunt-ended, Gram-negative bacilli. Single colonies were picked for PCR detection. The results showed that the virulence genes of the isolate from the brain tissue of infected dead mice were consistent with those of the isolate in this experiment, indicating that this isolate could cross the blood–brain barrier and enter brain tissue (Figure 11 and Figure 12). Microscopic histopathological examination (H&E staining, 100×) provided further evidence of severe tissue damage. The brain tissue exhibited vascular congestion in the meninges, widespread neuronal necrosis (characterized by pyknosis and karyorrhexis), mild glial cell proliferation, neuronal edema, and the formation of micro-vacuoles in the interstitium (Figure 13). Additionally, marked pathological lesions, including hemorrhage, inflammatory cell infiltration, and cellular necrosis, were observed across the lungs, liver, spleen, and heart (Figure 14).

### 3.5. Neuroinflammatory Response in Brain Tissue

**Figure 15:**
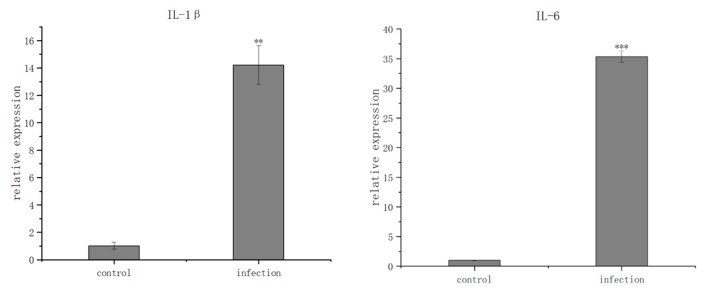

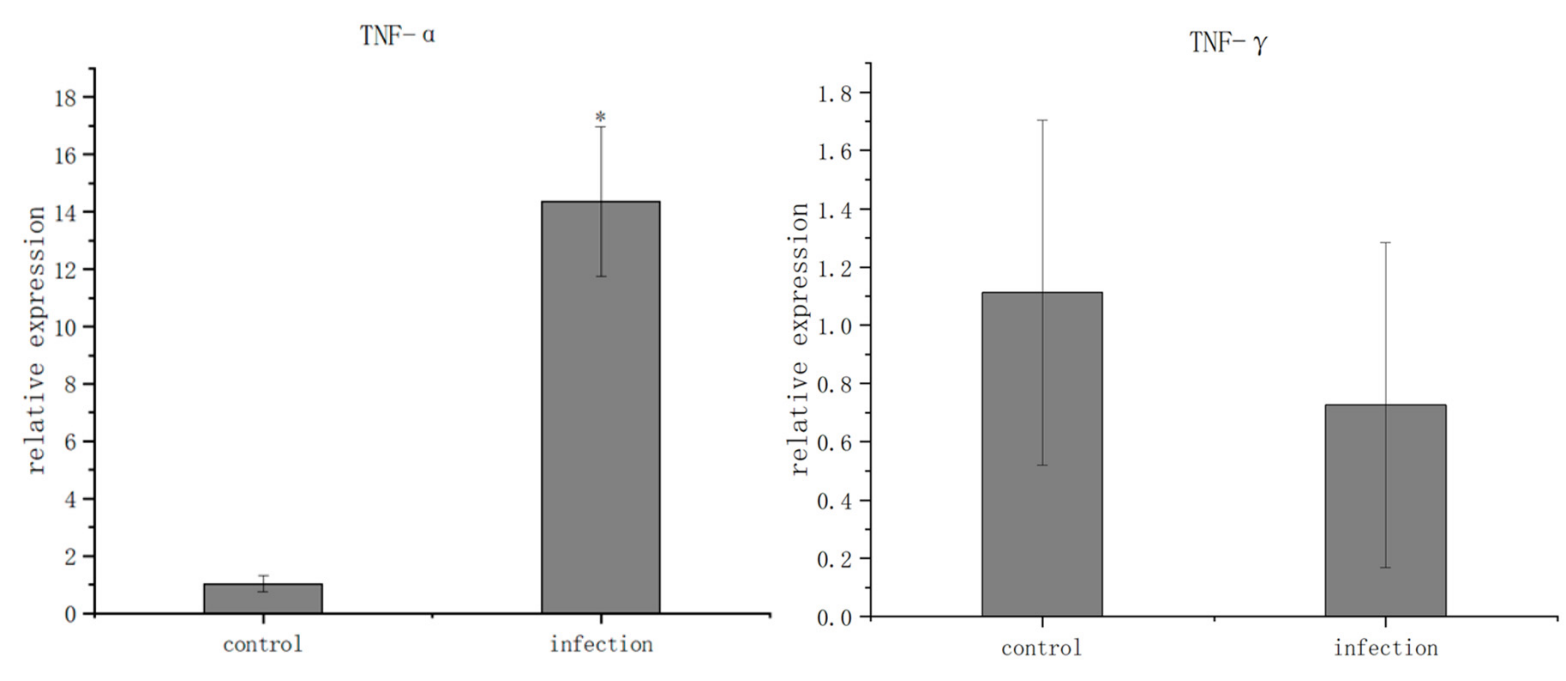
Neuroinflammatory cytokine mRNA expression in murine brain tissue. Note: * *p* < 0.05 indicates that the data have significant differences; **: *p* < 0.01 indicates that the data show highly significant differences; ***: *p* < 0.001 indicates that the data have extremely significant differences.

To assess the host immune response to the NMEC infection, the mRNA expression levels of key inflammatory cytokines in the brain tissue were quantified via RT-qPCR. Compared to the negative control group, the transcription levels of pro-inflammatory cytokines were dramatically upregulated in the infected mice. Specifically, the relative mRNA expression of IL-1β surged from 1.02 to 14.22 (*p* < 0.01), IL-6 skyrocketed from 1.00 to 35.35 (*p* < 0.001), and TNF-α (referred to as TNF-α in the original text, assumed TNF-α based on context) increased from 1.02 to 14.37 (*p* < 0.05). Conversely, the expression of IFN-γ showed a slight but non-significant decrease from 1.11 to 0.73 (*p* > 0.05). These robust molecular signatures of severe neuroinflammation further substantiate that the isolated *E. coli* isolate successfully induced acute meningitis in the murine model (Figure 15).

## 4. Discussion

ExPEC represents a highly adaptable and diverse group of pathogens, encompassing UPEC, NMEC, SEPEC, and APEC [33]. Unlike intestinal pathotypes, ExPEC strains lack universally conserved virulence-associated genes (VAGs) and are typically classified based on their clinical manifestations and isolation sources [34]. In this study, the bacterial strain was isolated from the brain tissue of a calf suffering from acute meningitis. However, in the in vivo murine model, the isolate induced profound clinical signs and pathological lesions characteristic of both meningitis and systemic sepsis. This strongly suggests that the isolate is a hybrid pathotype, simultaneously exhibiting NMEC and SEPEC characteristics. More than 50 VAGs—encoding adhesins, toxins, protectins, siderophores, and invasins—have been implicated in ExPEC pathogenicity. These VAGs are frequently located on mobile genetic elements (MGEs), which facilitate horizontal gene transfer among different bacterial populations [35]. This genetic exchange drives the emergence of hybrid strains with augmented virulence [36]. In the abdominal cavity infection model, this strain demonstrated the ability of systemic dissemination and brain tissue colonization, suggesting its potential to cross the BBB. Subsequently, the neural invasion mechanism under natural infection needs to be further verified through the gut–brain axis model. The multi-pathotype nature of such hybrid *E. coli* strains complicates traditional classification systems and clinical diagnostics [37]. By retaining the capacity to infect diverse intra- and extraintestinal niches, hybrid strains complicate therapeutic interventions and significantly increase the risk of severe, systemic disease [38].

Epidemiological studies have mapped the diverse ST distribution of ExPEC isolates, with lineages such as ST131, ST73, ST95, and ST69 comprising 38% to 61% of clinical isolates globally [39]. Specifically, ST131 consistently dominates in bloodstream infections, whereas NMEC is most frequently associated with ST95 and ST1193 [40]. Notably, the isolate belongs to ST1434 [41]. Furthermore, while the majority of highly virulent ExPEC strains are assigned to phylogroups B2 and D, our isolate belongs to phylogroup A [42]. This aligns with recent findings reported that beef-derived ExPEC isolates were predominantly distributed in phylogroup A (64.3%), diverging from the typical human clinical patterns [43]. Classically, NMEC strains heavily rely on the K1 capsule and specific O-antigens for immune evasion, with serotypes like O18:K1:H7, O1:K1, O7:K1, and O45:K1:H7 accounting for over 70% of NMEC cases, and approximately 80% expressing the K1 capsule [44]. In stark contrast, our isolate (O18ab:H14) completely lacks a capsule yet encodes an extensive array of adhesins and iron-acquisition systems. This significant deviation from traditional NMEC paradigms suggests the presence of alternative, highly efficient compensatory mechanisms for host adaptation and immune evasion.

WGS has revolutionized the evaluation of both the core and accessory genomes of *E. coli*, establishing itself as the gold standard for strain characterization [45]. The application of bioinformatic tools is crucial for identifying VFs and unraveling their intricate relationships with hypervirulent phenotypes. Our genomic analysis identified 202 VFs encompassing diverse functional categories: immunomodulation (26 genes), adhesins (41), biofilm formation (8), motility (54), exotoxins (1), invasion (4), regulation (8), effector delivery systems (33), antimicrobial competition (4), and nutritional/metabolic factors (22). The high prevalence of genes such as *nlpI* and *fimH* aligns with previous ExPEC studies, underlining their critical roles in enhancing host fitness. Fimbriae (e.g., *fimH*) are pivotal for the initial attachment to host cells—a prerequisite for colonization, biofilm formation, and immune evasion [46]. Concurrently, biofilm-associated genes (*csgA*, *aslA*) bolster the pathogen’s resilience against host defenses and antimicrobial agents. Robust iron-acquisition systems (*entA*, *entB*, *entE*) empower the bacteria to thrive in iron-depleted host environments like the bloodstream, while immunomodulatory genes (*rfbA*, *rmlB*, *ugd*, *rfaE*) promote serum resistance and tissue persistence, potentially compensating for the lack of a physical capsule. Crucially, the isolate harbors *ibeB*, *ibeC*, and *ompA*, which are extensively documented as essential determinants for penetrating the BBB in NMEC infections. The broad virulence profile observed here mirrors recent reports indicating a vast diversity of VAGs in bovine ExPEC, including the presence of Type VI and Type IV secretion systems in mastitis-associated strains [47]. However, the annotation of virulence genes based on VFDB only suggests potential functions and cannot confirm their actual expression or compensatory effects. In the future, methods such as gene knockout and transcriptome analysis are needed to verify the specific roles of these genes in the phenotype of capsule deficiency.

The proliferation of multidrug-resistant *E. coli* (MDR-E) poses a monumental risk to global public health, significantly elevating morbidity and mortality rates associated with severe infections like sepsis, meningitis, and UTIs [48]. ExPEC strains are frequently heavily armed with antimicrobial resistance genes (ARGs), leading to widespread treatment failures in both human and veterinary medicine, while accelerating the dissemination of AMR [49]. Our isolate exhibited a profound MDR phenotype, underpinned by 73 strictly defined ARGs operating through seven distinct mechanisms: antibiotic target alteration (60 genes), antibiotic efflux (93), antibiotic inactivation (4), target protection (2), reduced permeability (13), resistance by absence (4), and target replacement (2). However, the detection of drug-resistant genes does not necessarily correspond to the phenotype. In the future, comprehensive evaluation still needs to be combined with expression regulation and protein function analysis. Despite the immense socio-economic burden imposed by ExPEC, variant and hybrid pathogenic strains in veterinary sectors are often critically under-monitored [50]. From an evolutionary perspective, the emergence of this new hybrid strain may be the result of the adaptive evolution of bacteria under long-term antibiotic pressure and ecological environment changes. This suggests that in the veterinary field, we need to pay more attention to the evolutionary dynamics of bacteria so as to take effective prevention and control measures in a timely manner. We need to emphasize the importance of strengthening biosecurity measures on farms, establish a sound monitoring system, and detect and handle the emergence of drug-resistant strains in a timely manner.

## 5. Conclusions

Overall, these findings underscore that the richness and diversity of virulence and resistance profiles across different *E. coli* STs and sample origins are far more extensive than previously recognized. The isolation of a highly lethal, capsule-deficient, MDR NMEC/SEPEC hybrid strain (O18ab:H14, ST1434) from a calf emphasizes the critical need for continuous genomic surveillance. Future research must prioritize comparative genomics, robust epidemiological models, and functional in vivo analyses to identify unique virulence signatures, elucidate their exact molecular mechanisms, and ultimately strengthen One Health strategies to control the dissemination of these formidable ExPEC variants. In the future, further in-depth research should be conducted on the pathogenic mechanism and transmission rules of this new hybrid strain, and more effective diagnostic methods and prevention and control strategies should be developed.

## Figures and Tables

**Table 1:** PCR primers for detection of *E. coli* species-discriminatory gene and 16s rRNA region.

Target	Primer Sequences (5′-3′)	Annealing Temperature/°C	Product Size/bp
*gapA*	CCGGCTAACCTGAAATGGGA	55	272
	CAACTTCGTCCCATTTCAGG		
16s rRNA	AGAGTTTGATCCTGGCTCAG	55	1500
	GGTTACCTTGTTACGACTT		

**Table 2:** Primers for detection of virulence-associated genes.

Genes	Primer Sequences (5′-3′)	Annealing Temperature/°C	Product Size/bp
*ompA*	ACGGTTCCGACTACTCTGCT	55	79
*fimH*	CCGTCAACGTCCGTCCAAAT	60	438
	ATGTAAACCTTGCGCCCGTC		
	CACGAGCAGAAACATCGCAG		
*nlpI*	GCCCAGGAGCGATAATTCCA	60	88
	CACGGCACTGTTCAAACTGG		
*malX*	AACAGCACCGAAAGTCACGC	130	60
	GACATCATGGCTGCTAAGAGAAC		

**Table 3:** Primer sequences for immune factor detection via RT-qPCR.

Target	Primer Sequences (5′-3′)
IL1β-F	TGCCACCTTTTGACAGTGATG
IL1β-R	TGATGTGCTGCTGCGAGATT
IL6-F	GGGACTGATGCTGGTGACAA
IL6-R	ACAGGTCTGTTGGGAGTGGT
TNFα-F	TAGCCCACGTCGTAGCAAAC
TNFα-R	TGTCTTTGAGATCCATGCCGT
IFN-γ-F	GGAGGAACTGGCAAAAGGATG
IFN-γ-R	GTTGCTGATGGCCTGATTGT
Actinβ-F	TTCTTTGCAGCTCCTTCGTT
Actinβ-R	ATGGAGGGGAATACAGCCC

**Table 4:** Identification of the biochemical characteristics of the isolated bacteria.

Strain	Biochemical Test	Result
*E. coli*	MR-VP	−
	Glucose	+
	Sucrose	+
	Lactose	+
	Mannitol	+

**Table 5:** The result of the antibiotic susceptibility test for the bacteria isolate.

Name of Antibiotic	Diameter of Antibacterial Zone (mm)	Judgment of Sensitivity
Amoxicillin	0	R
Gentamicin	12	R
Tetracycline	13	R
Polymyxin	10	I
Streptomycin	8	I
Enrofloxacin	14	I
Ciprofloxacin	13	R
Ofloxacin	15	I
Rifampicin	7	R
Cefepime	15	R

Note: R = resistant, I = intermediate, S = susceptible. Interpreted according to CLSI M100 2024 [30].

## Data Availability

The original contributions presented in this study are included in the article/Supplementary Material. Further inquiries can be directed to the corresponding authors.

## Supplementary Materials

The following supporting information can be downloaded at https://www.mdpi.com/article/10.3390/vetsci13040401/s1, Table S1: Predicted drug resistance genes of isolated bacteria; Table S2: Comprehensive profile of predicted virulence factors and antimicrobial resistance genes in the isolated *E. coli* strain O18ab:H14.
